# Effective dose of propofol combined with intravenous esketamine for smooth flexible laryngeal mask airway insertion in two distinct age groups of preschool children

**DOI:** 10.1186/s12871-024-02421-z

**Published:** 2024-02-05

**Authors:** Bin Zhang, Mingzhuo Li, Yuejiao Han, Xianliang Zhao, Chunhong Duan, Junxia Wang

**Affiliations:** 1 Department of Anesthesiology, Jinan Children’s Hospital (Qilu Children’s Hospital of Shandong University), Jinan, 250000 China; 2 School of Pharmacy, Linyi University, Linyi, 276000 China; 3 Center for Big Data Research in Health and Medicine, The First Affiliated Hospital of Shandong First Medical University & Shandong Provincial Qianfoshan Hospital, Jinan, 250000 China; 4 Department of Anesthesiology, Jinan Children’s Hospital (Qilu Children’s Hospital of Shandong University), Jinan, 250000 China; 5 Department of Pediatrics, Jinan Children’s Hospital (Qilu Children’s Hospital of Shandong University), Jinan, 250000 China; 6 Department of Pediatrics, The First Affiliated Hospital of Shandong First Medical University & Shandong Provincial Qianfoshan Hospital, Jinan, 250000 China

**Keywords:** Propofol, Esketamine, Effective dose, FLMA, Children

## Abstract

**Background:**

There is limited research on the combined use of propofol and esketamine for anesthesia induction during flexible laryngeal mask airway (FLMA) in pediatric patients, and the effective dosage of propofol for FLMA smooth insertion remains unclear. We explored the effective dose of propofol combined with intravenous esketamine for the smooth insertion of FLMA in two distinct age groups of preschool children.

**Methods:**

This is a prospective, observer-blind, interventional clinical study. Based on age, preschool children scheduled for elective surgery were divided into group A (aged 1–3 years) and group B (aged 3–6 years). Anesthesia induction was started with intravenous administration of esketamine (1.0 mg.kg^− 1^) followed by propofol administration. The FLMA was inserted 2 min after propofol administration at the target dose. The initial dose of propofol in group A and group B was 3.0 mg.kg^− 1^ and 2.5 mg.kg^− 1^, respectively. The target dose of propofol was determined with Dixon’s up-and-down method, and the dosing interval of propofol was 0.5 mg.kg^− 1^. If there was smooth insertion of FLMA in the previous patient, the target dose of propofol for the next patient was reduced by 0.5 mg.kg^− 1^; otherwise, it was increased by 0.5 mg.kg^− 1^. The median 50% effective dose (ED_50_) for propofol was estimated using Dixon’s up-and-down method and Probit analysis, while the 95% effective dose (ED_95_) was estimated through Probit analysis. Vital signs and adverse events during induction were recorded.

**Results:**

Each group included 24 pediatric patients. Using Dixon’s up-and-down method, the ED_50_ of propofol combined with esketamine for smooth insertion of FLMA in group A was 2.67 mg.kg^− 1^ (95%CI: 1.63–3.72), which was higher than that in group B (2.10 mg. kg^− 1^, 95%CI: 1.36–2.84) (*p* = 0.04). Using Probit analysis, the ED_50_ of propofol was calculated as 2.44 (95% CI: 1.02–3.15) mg.kg^− 1^ in group A and 1.93 (95% CI: 1.39–2.32) mg.kg^− 1^ in group B. The ED_95_ of propofol was 3.72 (95%CI: 3.07–15.18) mg.kg^− 1^ in group A and 2.74 (95%CI: 2.34–5.54) mg.kg^− 1^ in group B. In Group B, one pediatric patient experienced laryngospasm.

**Conclusion:**

The effective dose of propofol when combined with intravenous esketamine for smooth insertion of FLMA in children aged 1–3 years is 2.67 mg.kg^− 1^, which is higher than that in children aged 3–6 years (2.10 mg. kg^− 1^).

**Trial registration:**

Chinese Clinical Trial Registry Center (Registration Number: ChiCTR2100044317; Registration Date: 2021/03/16)

## Background

Laryngeal mask airway (LMA) is commonly used for airway management during pediatric anesthesia, and compared with tracheal intubation, it has the advantages of low risk of anesthesia complications, short anesthesia time, and relatively small airway stimulation. LMA is especially suitable for relatively short surgery and can avoid the administration of muscle relaxants during anesthesia [1, 2]. The flexible laryngeal mask airway (FLMA) has reliable ventilation and low airway adverse reactions and can be easily inserted into the pharynx, even without muscle relaxants [3]. In comparison to the classic LMA, the FLMA tube is more flexible and longer, enabling increased movement without the need for cuff rotation or compromising the seal against the larynx [4]. However, airway reflex caused by pharyngeal irritation during FLMA insertion may lead to FLMA insertion failure or displacement, which is associated with insufficient anesthetic depth. Therefore, appropriate anesthesia depth, which also reduces airway reflexes resulting from pharyngeal irritation, is a determining factor for improving the success rate of FLMA insertion [5, 6].

Intravenous anesthesia induction is the most commonly used induction method. Compared with inhalation induction, intravenous induction can reduce the incidence of perioperative adverse events in high-risk pediatric patients with respiratory distress [7], while avoiding the fear caused by the mask during inhalation induction and the impact of the pungent smell of inhaled anesthetics on pediatric patients [8]. Propofol, a widely used intravenous anesthetic for induction, has a rapid onset, can effectively inhibit airway reflex, and is often used for LMA insertion. However, high doses of propofol can lead to respiratory and cardiovascular depression [6]. Although the combination of propofol and opioids can reduce the dosage of propofol during LMA placement, it also increases the incidence of apnea [9]. Esketamine, an S-enantiomer of ketamine, is twice as potent as racemic ketamine, which can achieve more reliable sedation and analgesia with a relatively low risk of psychotomimetic and cognitive adverse effects than racemic ketamine [10]. It can maintain airway tension and hemodynamic stability and is an ideal anesthetic induction agent [11, 12]. In addition, low-dose esketamine may reduce the incidence of anesthesia-related respiratory depression by increasing ventilatory CO_2_ sensitivity [13]. In pediatric studies, esketamine has been shown to reduce postoperative pain in children [14] and decrease the occurrence of emergency agitation [15]. Currently, the safety and efficacy of propofol in combination with esketamine for sedation and gastroscopy in children have been reported [16, 17]. However, there is no clear evidence of the effective dose of propofol in combination with esketamine for FLMA insertion during anesthesia induction in children.

Many studies use the age of 3 as the criteria for grouping and selecting study participants [18–20]. Additionally, these studies have verified the variations in effective doses of intravenous anesthetics among pediatric patients in different age groups [18–20]. Propofol, being lipophilic, has its distribution volume associated with the body’s fat content. The fat content in children decreases progressively with age, potentially impacting the effective dose of propofol [12]. Consequently, the present study narrowed down the age range and specifically enlisted preschool children as study participants. Moreover, we conducted a comparison between preschool children under 3 years old and those over 3 years old, without the utilization of muscle relaxants, to ascertain the effective propofol dose required for the smooth insertion of FLMA in combination with esketamine. We hypothesize that the effective dose of propofol for smooth insertion of FLMA may vary in preschool children of different ages, and the effective dose of propofol for children younger than 3 years old may be higher than that for preschool children older than 3 years old.

## Methods

### Study design and ethics

This is a prospective, observer-blind, interventional clinical study. It was registered before patient enrollment at the Chinese Clinical Trial Registry Center (Registration Number: ChiCTR2100044317; Registration date: 16/03/2021). This study was approved by the Ethics Committee of the Qilu Children’s Hospital of Shandong University (IRB: QLET-ITB/P-2,021,031). All methods were performed following the Declaration of Helsinki. Written informed consent was obtained from the parents or other legal guardians of all enrolled children participating in the trial.

### Participants

Pediatric patients who were scheduled for elective surgery at the Qilu Children’s Hospital of Shandong University from March 2021 to July 2022 were recruited. Inclusion criteria: (1) preschool children aged from 1 to 6 years old; (2) patients with American Society of Anesthesiologists (ASA) physical status of I or II; (3) patients who received minor surgeries (such as skin lumpectomy, debridement, and suturing, and injection therapy for skin hemangioma) and had spontaneous breathing under FLMA ventilation and general anesthesia. Exclusion criteria: (1) patients with body weight less than 10 Kg; (2) obese patients with body mass index > 35 kg. m^− 2^; (3) patients with reactive airway disease; (4) patients with evident difficult airway (Mallampati III or IV, micro mandible, limited mouth opening, limited neck movement, etc.); (5) patients with obstructive sleep apnea-hypopnea syndrome; (6) patients with risk of gastroesophageal reflux; (7) patients with major organ diseases (such as kidney, liver, and heart diseases); (8) patients with abnormal laboratory examination results; (9) patients who were allergic to either propofol or esketamine. Based on the inclusion and exclusion criteria, pediatric patients were randomly selected. Based on the cut-off age of 3 years, the enrolled children were grouped into Group A (age older than 1 year old and younger than or equal to 3 years old) and Group B (age older than 3 years and younger than 6 years).

### Anesthesia protocol

A standardized anesthetic regimen was used in all participants. No premedication was administered. Peripheral venous access was made in the ward. All patients fasted for at least 6 h for solids and 2 h for clear fluids and were transferred to the operating room. The electrocardiography, noninvasive blood pressure, and peripheral oxygen saturation (SpO_2_) of each patient were monitored. Children were placed in a supine position with a thin pillow under the shoulder, and the head was kept slightly backward to maintain airway patency. Pure oxygen was inhaled and the oxygen flow rate was 2 L/min by mask ventilation to ensure oxygen supply during anesthesia induction. Glycopyrrolate (5 μg.kg^− 1^) and ondansetron (0.1 mg.kg^− 1^) were intravenously administrated to reduce oral secretions and the risk of postoperative vomiting, respectively. Then, anesthesia induction was initiated by intravenous administration of esketamine 1.0 mg.kg^− 1^ (lasting more than 30 s), followed by slow administration (more than 1 min) of the target dose of propofol. At 2 min after the propofol injection, the mask was gently removed and mask ventilation was terminated. The FLMA of corresponding sizes was inserted using the standard method. The size of FLMA was selected according to children’s body weight: size 2.0 for 10–20 Kg and 20 Kg; and, size 2.5 for 20-30Kg. The FLMA cuff was deflated and lubricated dorsally with water-based jelly. During FLMA insertion, the children were kept in a neck-flexed-and-head-extended position. The tip of the FLMA was pressed against the hard palate, and the FLMA was slowly pushed back along the midline of the palate. The insertion of FLMA was stopped when resistance was felt, and then the anesthesia machine was connected with a semi-open anesthesia circuit. The FLMA cuff was inflated until the cuff pressure reached 40 cmH_2_O according to a manometer. The respiratory rhythm of the children and the waveform of end-tidal CO_2_ on the anesthesia machine were immediately monitored for 1 min. When stable spontaneous breathing was achieved, the FLMA was fixed. All FLMA insertion was performed by the same anesthesiologist with experience in FLMA insertion more than 500 times.

During and after the insertion of FLMA, if there were minimal physical movements or weak hemodynamic fluctuations, additional propofol (2 mg.kg^− 1^) was slowly injected. If there were significant physical movements or hemodynamic fluctuations, the FLMA was immediately removed. Subsequently, anesthesia was deepened by administering inhaled sevoflurane through a mask before reinserting the FLMA.

Anesthesia was maintained with a combination of inhaled sevoflurane and fentanyl. Spontaneous breathing was maintained during surgery. The body temperature of all pediatric patients was continuously monitored during surgery, and blankets were used to maintain body temperature in non-surgical areas. After the surgery, the FLMA was removed and the children were transferred to the post-anesthesia care unit for monitoring.

### Data records

Heart rate (HR) and mean arterial pressure (MAP) before induction (baseline) were recorded by the anesthesiologist performing the FLMA insertion. HR and MAP before FLMA insertion, and at 1 min after FLMA insertion were recorded by a different anesthesiologist who was blinded to study grouping and drug administration. The blinded anesthesiologist was not present in the operating room before induction and was called into the operating room by a nurse immediately after the administration of propofol, to monitor the anesthesia induction. The blinded anesthesiologist also observed the response of children during FLMA insertion, the respiratory condition at 1 min after insertion, and any adverse events that occurred during induction.

Based on previous studies [21, 22], smooth insertion of FLMA was defined as follows: the FLMA was easily inserted without significant resistance; there was no physical movement, coughing, swallowing, breath holding, apnea, and laryngospasm; there was stable spontaneous breathing at 1 min after connecting the anesthesia machine; and the fluctuation of HR and MAP during and at 1 min after FLMA insertion was less than 20% of those before insertion. Otherwise, it was considered an unsmooth FLMA insertion.

Adverse events during induction were recorded: hypoxemia (SpO_2_ < 90% for more than 1 min), hypotension (MAP below 20% of baseline), tachycardia (HR more than 180 beats/min), bradycardia (for children aged 1–3 years: HR less than 80 beats per min; for children aged 3–6 years: HR less than 65 beats per min) [23]. The adverse events during and after surgery were also recorded.

### Dixon’s up-and-down method

Following intravenous administration of esketamine, the target dose of propofol was determined by the response of the previous patient to the insertion of FLMA using Dixon’s up-and-down method [24]. The initial dose of propofol administered to the first child aged 1–3 years and 3–6 years was 3.0 and 2.5 mg.kg^− 1^, respectively, and the step size of propofol was 0.5 mg.kg^− 1^. If FLMA was inserted smoothly in the previous child, the target dose of propofol in the subsequent child was set at 0.5 mg.kg^− 1^ lower than the previous child. If there was unsmooth FLMA insertion in the previous child, the target dose of propofol in the subsequent child was set at 0.5 mg.kg^− 1^ higher than the previous child. A single dose was obtained from each patient, and the sequence was continued until seven crossover pairs (unsmooth FLMA insertion to smooth FLMA insertion) were reached in each group.

### Sample size

Dixon’s methodology recommends continuing the experiment until a minimum of four crossovers are reached [24, 25]. Paul et al. revealed that the inaccuracy of Dixon’s up-and-down method was minimized as the number of crossover pairs increased, but the improvement diminished as the number of crossover pairs exceeded six [26]. According to a similar study [25], our study enrolled patients until seven crossover pairs were obtained.

### Statistical analysis

All data were processed by IBM SPSS Statistics 26.0 (IBM SPSS Statistics for Windows, Version 26.0, Armonk, NY, IBM Corp; 2017). A 2-sided *p*-value less than 0.05 was considered statistically significant. Continuous variables with normal or non-normal distribution were presented as the mean ± standard deviation (SD) or median (interquartile range), respectively. For analysis of the HR and MAP recorded at various time points within the group, repeated-measures ANOVA with Bonferroni correction was used. According to Dixon’s up-and-down method [24], the ED_50_ of propofol enabling smooth FLMA insertion was determined by calculating the mean of the midpoint doses of seven independent pairs of children who experienced a crossover from unsmooth insertion to smooth insertion. The ED_50_ values between groups were compared using Student’s t-test. The data were also assessed by probit regression analysis to obtain the ED_50_ and 95% effective dose (ED_95_) of propofol for smooth FLMA insertion.

## Results

### Baseline characteristics of participants

**Fig. 1:**
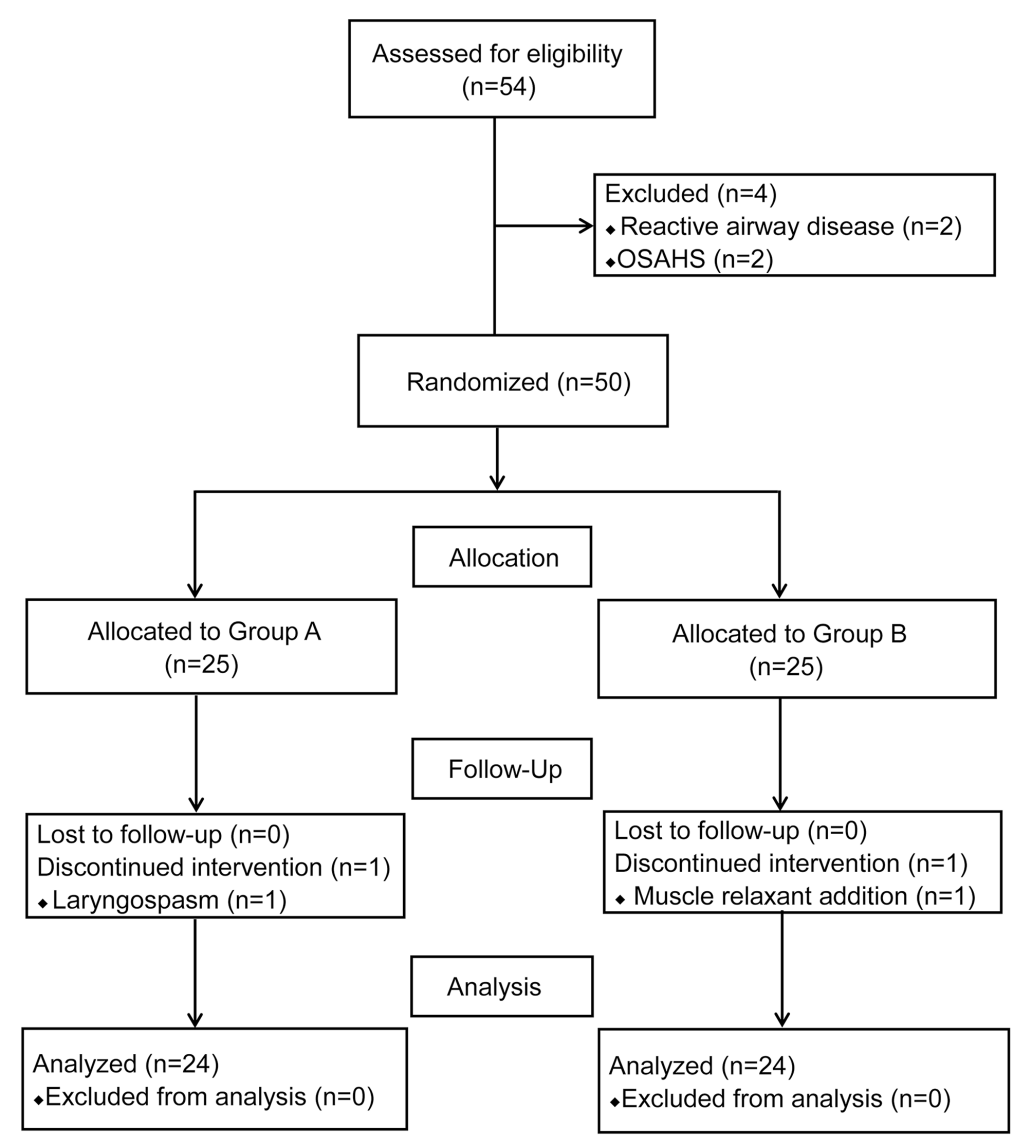
Flow diagram of patient recruitment

The flowchart for patient enrollment is shown in Fig. 1. A total of 54 children were initially recruited. After screening, 6 children were excluded, and 48 children were included in the final analysis, including 24 cases in Group A and 24 in Group B. The baseline characteristics are shown in Table 1.

**Table 1:** Baseline characteristics of patients

	Group A (*n* = 24)	Group B (*n* = 24)
Age (year)	2.1 ± 0.5	4.6 ± 0.7
Weight (kg)	13.9 ± 2.4	19.6 ± 4.1
Sex		
Males	56(%)	52(%)
Females	44(%)	48(%)
ASA Classification		
I	59(%)	52(%)
II	41(%)	48(%)

**Note**: American Society of Anesthesiologists. Data are expressed as mean ± standard deviation, or n (%), as appropriate

### The primary outcome: ED_50_ and ED_95_ of propofol for smooth FLMA insertion

**Fig. 2:**
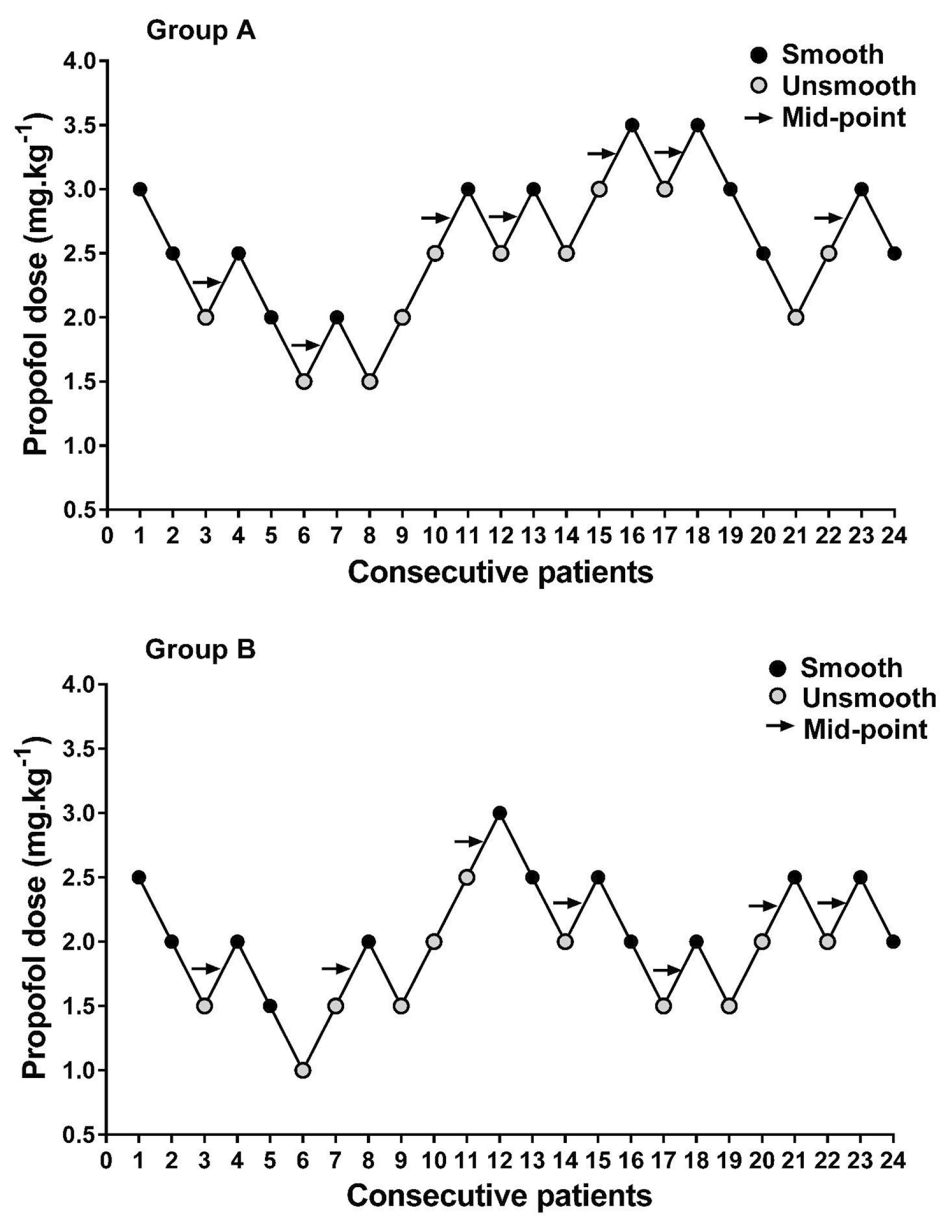
The sequential response of children in each group to FLAM insertion with Dixon’s up-and-down method. Arrows indicate the midpoint doses of all independent pairs of children involving a crossover

**Fig. 3:**
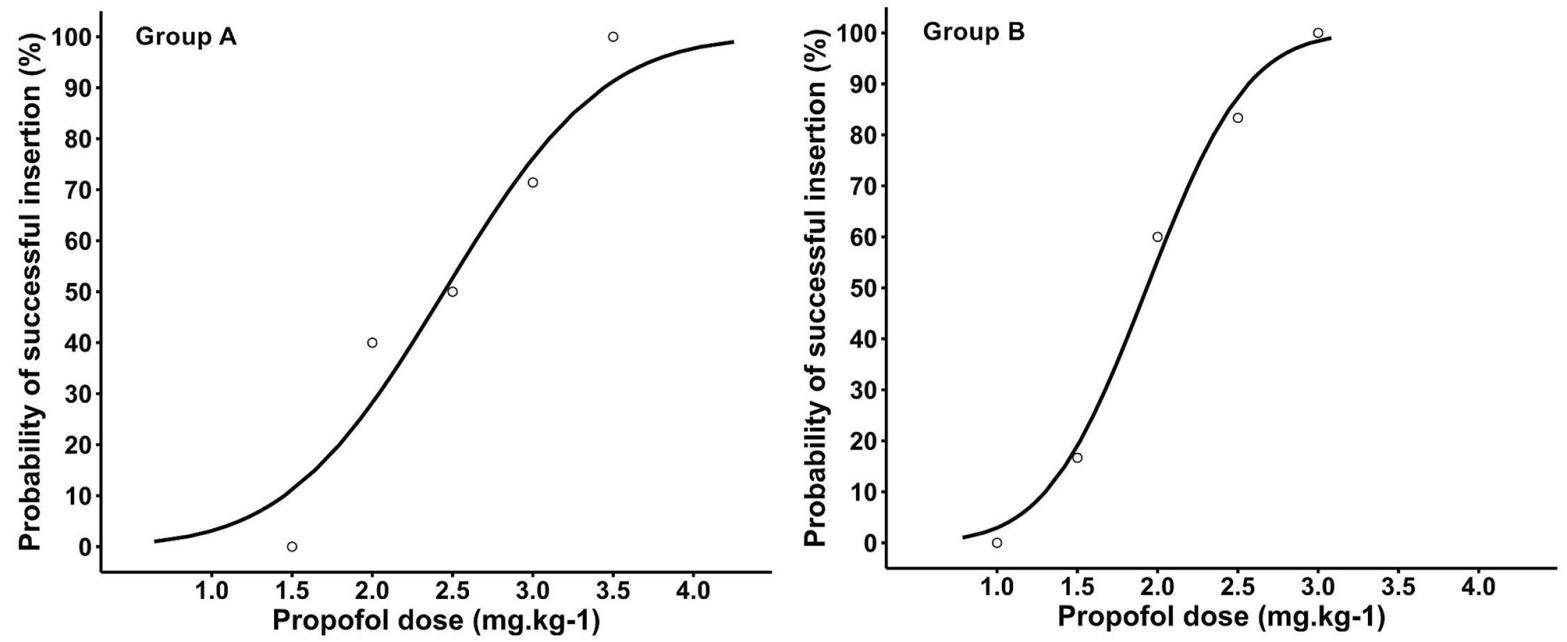
Dose-response curves of propofol combined with 1 mg.kg^− 1^ esketamine for FLMA insertion. The curves were plotted with Probit regression analyses

Using Dixon’s up-and-down method, the ED_50_ of propofol for smooth insertion of FLMA in group A was 2.67 (95% CI: 1.63–3.72) mg.kg^− 1^, which was significantly higher than that in group B (2.10, 95% CI: 1.36–2.84) mg.kg^− 1^ (*P* = 0.04) (Table 2). The sequences of smooth and unsmooth FLMA insertion in group A and group B are shown in Fig. 2. Probit regression analysis showed that the ED_50_ and ED_95_ of propofol for smooth insertion of FLMA in group A were 2.44 (95% CI: 1.02–3.15) mg.kg^− 1^and 3.72 (95% CI: 3.07–15.18) mg.kg^− 1^, respectively (Table 2). Meanwhile, the ED_50_ and ED_95_ of propofol in group B were 1.93 (95% CI: 1.39–2.32) mg.kg^− 1^ and 2.74 (95% CI: 2.34–5.54) mg.kg^− 1^, respectively (Table 2). The dose-response curve of propofol in each group is shown in Fig. 3.

**Table 2:** The effective dose of propofol for smooth insertion of FLMA in the two groups

	Group A (*n* = 24)	Group B (*n* = 24)	*P* value
**Dixon’s up-and-down method**			
ED_50_ (95%CI), mg.kg^− 1^	2.67 (1.63–3.72)	2.10 (1.36–2.84)	0.04
**Probit regression analysis**			
ED_50_ (95%CI), mg.kg^− 1^	2.44 (1.02–3.15)	1.93 (1.39–2.32)	--
ED_95_ (95%CI), mg.kg^− 1^	3.72 (3.07–15.18)	2.74 (2.34–5.54)	--

**Abbreviations**: FLMA, Flexible laryngeal mask airway. ED_50_, 50% effective dose; ED_95_, 95% effective dose

### The secondary outcomes

Hemodynamic parameters during induction at each time point are shown in Table 3. In group A, the HR at 1 min after FLMA insertion was significantly higher than that before FLMA insertion (*P* = 0.011). In group B, the HR at 1 min after FLMA insertion was significantly higher than the baseline value (*P* = 0.005) and that before FLMA insertion (*P* < 0.001). During anesthesia induction, there were no adverse events (such as tachycardia, bradycardia, and hypotension) in each group. One child in group A experienced transient hypoxemia (less than 1 min, minimum SpO_2_: 84%) resulting from weakened respiratory activity. After mandibular lifting and increased oxygen flow, hypoxemia was rapidly relieved, and spontaneous respiration was restored. One child in group B suffered laryngospasm after insertion of FLMA, which was relieved by the injection of propofol (2 mg.kg^− 1^) and mask ventilation without other additional intervention. Physical movement and increase of HR and MAP were the main manifestations of unsmooth FLMA insertion in both groups (Table 4).

All children remained stable during surgery. In the post-anesthesia care unit, one child in group A and one child in group B developed agitation. One child in group A had excessive oral secretions. After the adverse reactions disappeared, the patients were transferred to the ward. One child in group B had a sore throat. No other adverse reactions were observed.

**Table 3:** Heart rate and mean arterial pressure at each time point during induction

	Baseline	Before FLMA insertion	At 1 min after FLMA insertion
**Group A**			
Heart rate (beats min^− 1^)	115.2 ± 17.4	106.6 ± 17.0	119.2 ± 19.9^*^
Mean arterial pressure (mmHg)	67.2 ± 13.8	64.3 ± 13.3	66.2 ± 13.6
**Group B**			
Heart rate (beats min^− 1^)	101.1 ± 16.0	99.5 ± 12.3	111.1 ± 15.4 ^*#^
Mean arterial pressure (mmHg)	72.7 ± 12.5	72.2 ± 11.4	74.8 ± 10.1

**Note**: Data are expressed as mean ± standard deviation. *P < 0.05 vs. Before insertion. ^#^*P* = 0.005 vs. Baseline

**Abbreviation**: FLMA, Flexible laryngeal mask airway

**Table 4:** The reaction of children caused by unsmooth FLMA insertion

Reaction of children	Group A (*n* = 24)	Group B (*n* = 24)
Physical movement	12	8
Breath holding	4	4
Coughing	1	0
Swallowing	2	1
Increased heart rate/mean arterial pressure ^Δ^	5	5
Laryngospasm	--	1

**Note**: ΔThe increase of heart rate and mean arterial pressure after FLMA insertion was above 20% than baseline

**Abbreviations**: FLMA, Flexible laryngeal mask airway

## Discussion

In this study, we used Dixon’s up-and-down method to determine the effective dose of propofol combined with intravenous administration of esketamine 1 mg.kg^− 1^ for smooth insertion of FLMA in two distinct age groups of preschool children. We found that the effective dose of propofol in children aged 1–3 years was higher than that in children aged 3–6 years.

Several studies have shown that the effective dose of anesthetics in children is age-related, and the younger the child, the greater the effective dose required [19, 27]. A previous study focusing on pediatric sedation in emergency departments showed that children under 6 years old required increased doses of ketamine for sedation than children over 6 years old, and children under 3 years old required more ketamine than children over 3 years old [19]. Khalila et al. showed that the older the children, the greater the amount of propofol needed for satisfactory sedation during gastroscopy [27]. Consistently, in this study, when combined with the same dose of esketamine, the effective dose of propofol for smooth insertion of FLMA was higher in children aged 1–3 years compared to those aged 3–6 years. This result was consistent with our hypothesis.

The distribution volume of drugs is an important determinant of the loading dose. Propofol is fat-soluble and its pharmacokinetics are affected by the fat content [28]. The fat content of children gradually decreases with the increase of age [18, 29]. Therefore, the higher distribution volume of propofol in children is the reason for higher loading doses of propofol in children than in adults [18]. The study by Lifshitz et al. [30] showed that the fat mass percentage of 2-6-year-old preschool children gradually decreased with age, although there were no significant statistical differences. Perhaps even slight changes in fat content in preschool children may lead to significant differences in the distribution volume, resulting in different effective doses of propofol for smooth insertion of FLMA in children aged 3–6 years compared to those aged 1–3 years. This could be the reason for the different effective doses of propofol in the two age groups observed in this study.

The ED_50_ is a common way to measure drug effects, while ED_95_ is more clinically relevant. Although Dixon’s up-and-down method is a commonly used approach to assess the ED_50_ of drugs, it is unable to obtain the ED_95_ [16]. Therefore, in this study, we used Probit regression analysis to determine the ED_95_ of propofol, which showed that the ED_95_ of propofol for smooth insertion of FLMA in children aged 1–3 years was also higher than that in those aged 3–6 years.

Although the administration of propofol (2.0-2.5 mg.kg^− 1^) alone can provide satisfactory conditions for LMA insertion in adults, there are also clinical manifestations caused by inadequate anesthesia depth, such as choking, limb movement, and swallowing [31]. In this study, considering the synergistic effect of propofol and esketamine on anesthesia depth during FLMA insertion, the initial dose of propofol in children aged 3–6 years old was set at 2.5 mg.kg^− 1^. Considering relatively higher fat content in children aged 1–3 years old and based on the results of our pilot study, the initial dose of propofol was set at 3.0 mg.kg^− 1^, which was higher than that in children aged 3–6 years old. We found that although the ED_95_ and initial dosage of propofol varied among different groups, the differences were not significant, especially for children aged 3–6 years, which further verified the effectiveness of the initial dose of propofol. In addition, the ED_50_ obtained by Probit regression analysis was close to but not the same as the ED_50_ obtained by Dixon’s up-and-down method, which has also been reported in other similar studies [32, 33].

Abedini et al. suggested that the appropriate time for LMA insertion with lower complications and rapid placement was 15 s after propofol administration [5]. In this study, FLMA insertion was performed at 2 min after the administration of propofol, but not 15 s. This difference may be explained by the following two reasons. Firstly, the drug regimen for anesthesia induction was different between our study and the study by Abedini et al. Abedini et al. used a combination of propofol, midazolam, and fentanyl for induction, which may induce anesthesia more rapidly and in a shorter time. Secondly, propofol takes effect in 40 s, and in this study, the FLMA was inserted 2 min after propofol administration to ensure the full effect of propofol. Meanwhile, the potential risks of esketamine combined with propofol on respiration and circulation could be observed during this time. In this study, there was no reported hypoxemia caused by prolonged asphyxia and no cardiorespiratory depression such as bradycardia and hypotension. The HR after FLMA insertion was higher than that before FLMA insertion, which was related to the design of this study. Although the stimulation of FLMA insertion resulted in increased HR, there was no tachycardia in both groups during anesthesia induction.

Esketamine can maintain hemodynamic stability during anesthesia induction [34, 35]. This study also showed that esketamine combined with propofol was a safe and effective regimen for anesthesia induction in children. Esketamine stimulates the sympathetic nerve system, which balances the cardiorespiratory depression caused by propofol. Although esketamine has less effect on the central respiratory drive, it can also produce respiratory depression when administered at high doses or rapidly [11]. In this study, we conducted slow injections of esketamine and propofol, which may also decrease the risk of respiratory depression.

In addition, we found that pretreatment with esketamine might alleviate the injection pain of propofol (data not shown). After the injection of propofol, children in each group did not show any reactions similar to pain stimuli such as involuntary limb movement of the injection site and rapid increase of HR. Fu et al. have also confirmed that the intravenous injection of low-dose esketamine (0.15 mg.kg^− 1^) in adults could reduce the incidence of propofol injection pain [36]. They considered that the main reason was the peripheral local anesthetic action of esketamine in the vascular endothelium, rather than the central analgesic effect.

There were several limitations in this study. Firstly, the anesthetic dose or depth of anesthesia for the insertion of different types of LMA is different [37, 38]. This study only focused on FLMA and may not be completely applicable to other types of LMA. Secondly, ketamine or esketamine can increase the bispectral index value of patients and thus interfere with the reliability of BIS [39]. Therefore, we did not use BIS to assess the depth of anesthesia when FLMA was inserted. To ensure an effective anesthesia depth, we used the maximum induction dose of esketamine. Thirdly, we did not use the target-controlled infusion to guide the propofol dose during induction. Although target-controlled infusion is becoming more and more popular among children, the accuracy of pharmacokinetic models in children still needs to be improved [18]. Finally, each group of children received identical doses of esketamine, which may have varying efficacy in children of different ages, thus impacting the effective propofol dosage. Therefore, in this study, the effective dose of propofol for each group of children was the effective dose when intravenously injected with 1.0 mg.kg^− 1^ of esketamine.

## Conclusion

The effective dose of propofol combined with intravenous esketamine for smooth insertion of FLMA was different in preschool children of different age groups. In detail, the effective dose of propofol in children aged 1–3 years was 2.67 mg.kg^− 1^, which was higher than that in those aged 3–6 years (2.10 mg. kg^− 1^). Propofol combined with esketamine is a safe and effective medication regimen for the smooth insertion of FLMA and can maintain hemodynamic and respiratory stability during FLMA insertion in preschool children.

## Data Availability

The datasets used and/or analyzed during the current study are available from the corresponding author upon reasonable request.

## List of abbreviations

ASA: American Society of Anesthesiologists
ED_50_: 50% effective dose
ED_95_: 95% effective dose
FLMA: Flexible laryngeal mask airway
HR: Heart rate
LMA: Laryngeal mask airway
MAP: Mean arterial pressure
SD: Standard deviation
SpO_2_: Peripheral oxygen saturation
